# A qualitative descriptive study on the perspectives and experiences of multidisciplinary HCPs in providing nutritional care to older adults with cancer

**DOI:** 10.1007/s00520-025-09254-7

**Published:** 2025-02-25

**Authors:** Rachel Li Yin Wong, Chad Yixian Han, Jolene Thomas, Reegan Knowles

**Affiliations:** 1 Nutrition and Dietetics, College of Nursing and Health Sciences, Flinders University, Sturt Road, Bedford Park SA5042, Adelaide, Australia; 2 Caring Futures Institute, College of Nursing and Health Science, Flinders University, Sturt Road, Bedford Park SA5042, Adelaide, Australia; 3 Department of Dietetics, National University Hospital, Singapore, Singapore

**Keywords:** Dietary advice, Dietetic referral, Nutrition education, Healthcare professionals, Older adults, Cancer

## Abstract

**Objectives:**

To explore the perceptions and experiences of healthcare professionals (HCPs) caring for older adults with cancer regarding dietary advice provision and dietetic referral.

**Methods:**

Qualitative descriptive study providing rich descriptions of the experiences of multidisciplinary HCPs in providing care to older adults with cancer, excluding dietitians or nutritionists. Purposive and snowball sampling methods were used for recruitment. Semi-structured interviews and a focus group session were conducted. Data was analysed using qualitative content analysis. Inductive codes were generated, and codes representing factors influencing HCPs’ referral to dietetics and dietary advice provision were then mapped to domains in the Tailored Implementation of Chronic Diseases (TICD) checklist using a deductive approach.

**Results:**

Twenty HCPs across various Australian healthcare settings participated, with a broad range of working experience (1.5 to 53 years) being interviewed. Most participants perceived their role in the provision of general dietary advice, and there was a consensus that nutrition is important. Key barriers (e.g. lack of time and resources, perceived lack of knowledge, scope of practice), including unique patient-related barriers (e.g. co-morbidities, fatalistic mindset), and key facilitators (e.g. need for education, evidence-based resources, multidisciplinary team approach) to dietary advice provision fell within five TICD domains (intervention, health professional factors, patient factors, professional interactions, incentives and resources). Common barriers (e.g. disconnections in multidisciplinary care, lack of structured referral pathways) and facilitators (e.g. clear referral pathways) to referral fell within four TICD domains (intervention, health professional factors, professional interactions, incentives and resources).

**Conclusion:**

The barriers and facilitators to referral and provision of dietary advice by HCPs suggest the need for system-level changes via a multi-pronged approach. Simple and accessible nutrition resources, stronger nutrition education for HCPs, improved referral pathways and role clarity are required to support a multidisciplinary approach to nutritional care. More qualitative research on patient-level factors involving older adults with cancer is warranted.

**Supplementary Information:**

The online version contains supplementary material available at 10.1007/s00520-025-09254-7.

## Introduction

Cancer survivorship rates have increased over the years due to earlier diagnosis, improved treatment and better survivorship care [1]. However, this rate decreases with increasing age [1]. Older adults (65 years and above) are more likely to develop cancer than the younger population [1]. They experience greater disease burden and poorer health outcomes due to other co-morbidities and age-related physical, emotional and psychological issues, increasing the complexity of their care [2].

Coupled with nutritional impairments due to the pathogenesis of cancer and its treatment, older adults face additional barriers to maintain an optimal nutritional status, which is a determinant of overall health [3, 4]. Although the causative relationship between malnutrition, cancer and ageing is yet to be fully elucidated, the detrimental impacts of malnutrition, including changes in body composition and weight loss, have been demonstrated to be far more pronounced amongst older people [5]. Malnutrition has been associated with poorer prognosis, lower survival rates and quality of life in older adults with cancer [6, 7]. It also contributes to other syndromes, such as sarcopenia and cachexia, which are interrelated and highly prevalent in older adults with cancer [5]. Additionally, malnutrition is strongly linked to the development of frailty, which further predisposes older adults to poorer treatment outcomes and a higher risk of complications and mortality [5, 8]. Therefore, the nutritional management of cancer, including nutritional assessment, dietary counselling, nutritional support and therapies, is critical in cancer care [9].

Individualised dietary counselling is effective in improving nutritional status in oncology patients, with several clinical guidelines including it as part of the standard of care [9, 10]. In the current Australian cancer care system, dietary counselling is mainly led by dietitians [11]. However, access to dietetic services differs across various healthcare settings, with greater challenges in rural areas due to geographical location and disproportionate funding for oncology-specific dietetic positions, placing a higher burden on patients [12, 13]. Additional barriers impacting access to dietetic services include inadequate referral pathways, suboptimal coordination of post-treatment nutrition care and cost of private oncology services [14–16]. This emphasises the importance of a multidisciplinary approach to nutritional care in clinical practice guidelines for people with cancer [17]. Apart from dietitians, other healthcare professionals (HCPs) may also contribute to nutritional care (including the provision of dietary advice) during routine care, particularly in situations where access to dietitians is suboptimal and dietary advice is general in nature. Despite strong evidence for a multidisciplinary approach, cancer survivors in Australia previously reported that non-nutrition HCPs do not provide adequate or appropriate dietary advice post-treatment [15]. Patients with cancer have previously indicated their desire for advice to be delivered by other HCPs as they value dietary guidance via a multidisciplinary approach throughout the continuum of their care [15, 16, 18].

Recent reviews in oncology found that although HCPs perceive nutrition care as part of their role, certain factors may influence their provision of dietary advice to older people, including a lack of knowledge and time [16, 18]. Although there is some understanding of dietary advice provision to people with cancer, there is limited research examining the views of HCPs regarding the provision of dietary advice and referral practices specifically to older people, which is likely to be different to younger adults. To facilitate the involvement of HCPs in the nutritional care of older people with cancer, a better understanding of HCPs’ perspectives is necessary. Hence, this research aimed to explore the perceptions and experiences of HCPs caring for older people with cancer regarding dietetic referral and provision of dietary advice, including their perceived barriers and facilitators to optimal nutritional care.

## Methods

### Study design and philosophical assumptions

The philosophical stance on the meanings of reality and knowledge should underpin all aspects of research, including choosing the methodology and data interpretation [19]. This research was underpinned by a relativist and subjectivist paradigm; researchers believe each participant’s experience contributes to the reality of a phenomenon. Therefore, the qualitative description methodology and focus group/interview methods were employed to meet the study aims [20] (Supplementary Material 1). The qualitative description approach aims to provide a comprehensive description of the phenomena by staying close to the data, aligning with the philosophical paradigm [21]. Moreover, this methodology is well-suited for issues that have limited previous research [21].

To obtain a comprehensive understanding of the issue, the Tailored Implementation for Chronic Diseases (TICD) checklist was used for further analysis of inductively identified factors influencing dietetic referral and dietary advice provision by HCPs [22]. This checklist can be used to identify factors influencing change in HCPs’ practice, which can inform the development of future interventions [22]. The TICD checklist has seven domains (Supplementary Material 2) and was selected based on the appropriateness of its purpose, specific use in patients with chronic diseases and inclusion of multiple domains [23].

### Data collection

#### Recruitment and participants

This study included HCPs who had not received formal training in nutrition or dietetics, worked in any healthcare setting across Australia and had current or past experiences providing care to older people with cancer. Recruitment occurred between July and November 2023 and was conducted through purposive and snowball sampling. Emails were sent to potential HCPs known to the research team via their professional networks. Potential participants were encouraged to forward the recruitment emails via their own professional networks. One researcher (RW) contacted HCPs who agreed to participate and screened them for eligibility. Attempts were made to recruit a range of professions to increase the diversity of perspectives [24]. Recruitment ceased once researchers perceived information power where adequate data was collected to answer the research aims [25]. This study was approved by the Southern Adelaide Clinical Human Research Ethics Committee (HREC reference: 104.22) and Flinders University Human Research Ethics Committee (FUHREC reference: 6379). All participants provided written informed consent. To ensure confidentiality and anonymity, each participant was provided with a numerical label (e.g. P1).

#### Interview protocol

A topic guide was developed by the research team to guide the semi-structured format of all interviews and focus group sessions (Supplementary Material 3). Initial questions sought to obtain information on participants’ past and present healthcare roles, work settings and their interactions with older people with cancer. Subsequent questions focused on five main areas regarding their perspectives on nutritional care in older people with cancer: (1) perceived importance of dietary advice on health outcomes, (2) their perceived roles, (3) current provision of dietary advice and referral to dietetics, (4) their awareness and use of dietary guidelines and (5) perceived barriers and facilitators to providing dietary advice.

#### Interviews and focus group session

All interviews were conducted in-person or online, according to participants’ preference, by one trained interviewer (RW). After the first three interviews, the recordings were checked by two researchers (RK, CH), and in-depth feedback was provided to RW to improve standardisation. The focus group was conducted by one researcher (RW), and CH was present to collect field notes. Participants were encouraged to share their thoughts freely with the interviewer, providing verbal prompts only where necessary to minimise interruptions to participants’ responses. All sessions were audio-recorded after obtaining participants’ verbal consent and transcribed verbatim (Microsoft Word, Version 2307). Transcripts were de-identified and cross-checked for accuracy by one author (RW) and imported into NVivo Software for coding [26].

### Data analysis

**Fig. 1:**
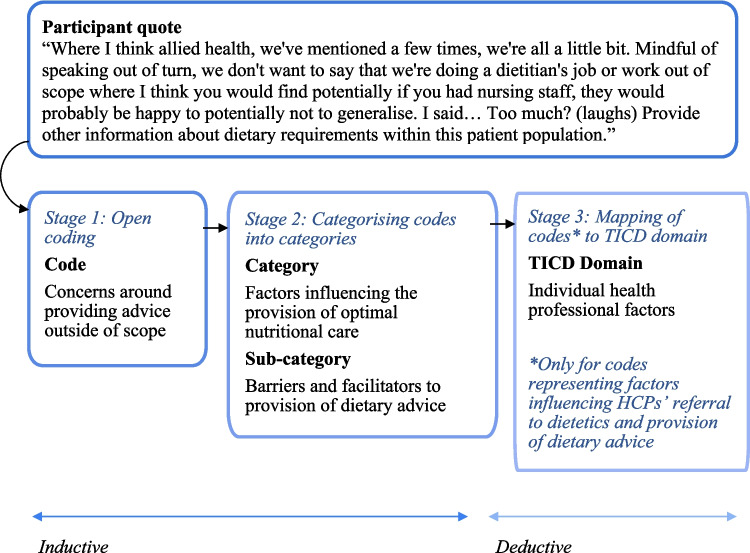
Three-stage data analysis process of healthcare professionals’ perspectives and experiences and an example of mapping a code representing a factor influencing healthcare professionals’ provision of dietary advice to a domain in the TICD

Data collection and analysis occurred simultaneously as per recommendations for qualitative research [27]. Participant characteristics were analysed using descriptive statistics and presented as frequencies and range. Qualitative content analysis was used to identify sub-categories and categories [28, 29]. The term ‘categories’ rather than ‘themes’ was chosen given the manifest level of abstraction during analysis [29]. The analysis process followed three main stages (see Fig. 1). Firstly, coding was conducted using an inductive approach, and open coding was done by the first author (RW). Codes were constructed to stay as close to the data as possible while keeping the research aims in mind. Two research team members (RK, CH) coded a subset of the transcriptions separately (15%). Coding discrepancies were discussed to reach a consensus. Second, codes were grouped into sub-categories and categories; a refined coding framework was developed after discussion with three other researchers (RK, CH, JT) Supplementary Material 4). Finally, to obtain a comprehensive understanding of the factors influencing dietetic referral and dietary advice provision by HCPs, RW employed a deductive approach to map the codes representing barriers and facilitators to domains in the TICD checklist [22]. The final analysis was reviewed by RK.

### Demonstrating positionality, reflexivity and rigour

This study was reported according to the Standards for Reporting Qualitative Research checklist [30] (Supplementary Material 5). All authors have backgrounds in nutrition and dietetics, including three trained dietitians with at least 10 years of dietetic/research experience each. The first author is a research student (RW) and was guided by co-authors who were researchers experienced in qualitative research [31–33]. There were no pre-existing relationships between the interviewer (RW) and participants. At the start of the interviews, RW introduced herself as a research student and explained the study objectives. RW acknowledged the influence of her dietetics background on her perspectives and employed reflexivity throughout the research. To confirm the findings, researcher triangulation was achieved through discussions with all authors. To enhance dependability, RW kept an audit trail of the coding iterations. Representative quotations were included to increase the confirmability and transferability of the findings (Supplementary Material 6).

## Results

**Fig. 2:**
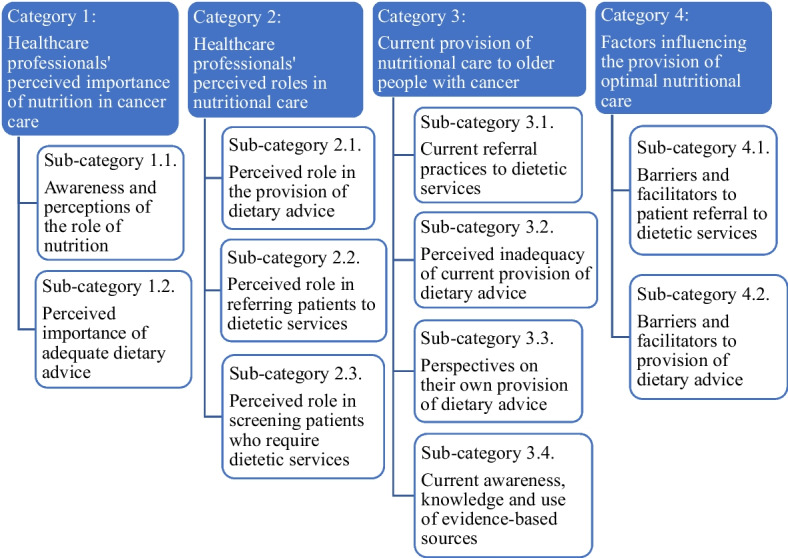
Healthcare professionals’ perspectives and experiences regarding referral practices and the provision of dietary advice to older people with cancer

Thirty HCPs were invited to consider participation via email between August and October 2023. Four were ineligible because they had no experience providing care to older persons with cancer. One HCP declined due to unavailability, and five did not respond to the recruitment email. A total of 20 HCPs were interviewed from August to November 2023: 16 individual interviews and one four-person focus group session, ranging between 22 to 47 min. The focus group session and two of the individual interviews were held in-person; all other interviews were online. Participant characteristics are presented in Table 1. A variety of healthcare professions working in Australia with a broad range of working experience (1.5 to 53 years) and work settings were interviewed. Four categories relevant to the research aims emerged during the analysis (Fig. 2).

**Table 1:** Summary of participant characteristics

Characteristics	Total *n* = 20
Profession, *n*	
Oncology team	
Medical oncologists	5
Oncology nurse practitioners	2
Medical doctors and other specialists	
Lymphedema specialist	1
Palliative care specialist	1
General medicine physician	1
General practitioner	1
Allied healthcare professionals	
Speech pathologists	2
Physiotherapists	2
Audiologist	2
Occupational therapist	1
Exercise physiologist	1
Pharmacist	1
Work setting, *n*	
Public hospital/ clinic	6
Private	3
Public and private	7
Research, public and private	4
Sex, *n*	
Female	6
Male	14
Years of work experience, *n*	
Less than 5 years	4
5–10 years	7
More than 10 years	9

### Category 1: HCPs’ perceived importance of nutrition in cancer care

Many participants perceived nutrition and dietary advice as important for older people with cancer, and participants were aware of the link between nutrition and health outcomes.

#### Sub-category 1.1. Awareness and perceptions of the role of nutrition

Most participants acknowledged the important role of nutrition in health and cancer-related outcomes for people with cancer. Many were aware of the higher risk of malnutrition in older people with cancer, especially those undergoing cancer treatments. Although one participant indicated the relationship between nutrition and cancer-related outcomes as overrated as they have ‘yet to see any evidence’ showing the direct link between nutrition and cancer outcomes, he still agreed that nutritional management and dietary advice are essential for comprehensive cancer care.

Other participants described the benefits of maintaining a good nutritional status, including improved energy levels, muscle function maintenance, frailty prevention and increased treatment tolerance.

#### Sub-category 1.2. Perceived importance of adequate dietary advice

Most participants agreed that it is important for older people to be provided with adequate dietary advice. Some participants provided reasons why dietary advice provision is important, while one participant said that the importance depends on the patient’s prognosis.

### Category 2: HCPs’ perceived roles in nutritional care

Participants identified their roles in nutritional care, including the provision of dietary advice, referral and screening of patients. Many participants elaborated on their supportive role to the dietitian in terms of advice provision, referral and screening.

#### Sub-category 2.1. Perceived role in the provision of dietary advice

Although there were diverse responses around their specific role in the provision of dietary advice, many participants agreed that all HCPs play a role. Several participants indicated that their role is to provide general advice and resources. Some participants (e.g. pharmacist, speech pathologist, lymphedema specialist) identified their role in providing advice specific to their profession. One participant stressed that HCPs can be more involved in providing dietary advice whenever there are opportunities, e.g. home visits.

#### Sub-category 2.2. Perceived role in referring patients to dietetic services

Many participants mentioned that their role is to refer patients to a dietitian whenever the advice they can provide is inadequate or when they feel that more specific advice is required.

#### Sub-category 2.3. Perceived role in screening patients who require dietetic services

Participants talked about their responsibilities in identifying nutritional problems in patients and picking up signs of malnutrition. Some participants discussed how their supportive role in screening is imperative.

### Category 3: Current provision of nutritional care to older people with cancer

Participants spoke about their perspectives on the current referral practices, adequacy of dietary advice and the quality of their provision of dietary advice, including perceived knowledge and use of evidence-based sources.

#### Sub-category 3.1. Current referral practices to dietetic services

Participants described the various reasons for referring older people with cancer to dietetic services. Many participants referred when they were concerned about the patient’s weight or risk of malnutrition. They also referred when they perceived that the patient required more specific advice.

Participants also reported on various referral pathways in different healthcare settings, including informal methods like email or messaging and formal referral process. Some participants identified that referral was a team effort and responsibility, while other participants highlighted that the responsibility should fall to one profession—nurses.

#### Sub-category 3.2. Perceived inadequacy of the current provision of dietary advice

The current provision of dietary advice to older people with cancer was perceived to be inadequate by most participants due to reasons including missed referrals, diet not being the main priority of some clinicians, poor accessibility to dietetic services and lack of dietitians. Participants highlighted certain patient groups that were at higher risk of receiving inadequate advice: older people and patients not on treatment. However, some participants mentioned that advice is adequate if patients are seeing a dietitian.

#### Sub-category 3.3. Perspectives on their own provision of dietary advice

The frequency of dietary advice provision varied amongst the different HCPs. While some reported that they generally do not give advice, nurses and oncologists were more likely to engage in advice provision. Many participants described the quality of their dietary advice as basic and informal advice, with one participant reflecting on how the advice provided was sub-optimal. Different information sources of nutritional knowledge were identified. Many participants mentioned that knowledge was from past work experiences, while some identified education sources like hospital grand rounds, nutrition webinars and basic nutritional lessons in school.

#### Sub-category 3.4. Current awareness, knowledge and use of evidence-based sources

Most participants reported on their poor awareness, knowledge and use of dietary guidelines for people with cancer. Some identified their existence but did not know the specific content. However, several participants discussed the use of evidence-based resources when providing advice.

### Category 4: Factors influencing the provision of optimal nutritional care

Participants highlighted various factors that influenced their referral practices to dietetics and the provision of dietary advice to older people with cancer. All inductively identified factors (codes) in this theme were mapped to a TICD domain.

#### Sub-category 4.1. Barriers and facilitators to patient referral to dietetic services by HCPs

**Fig. 3:**
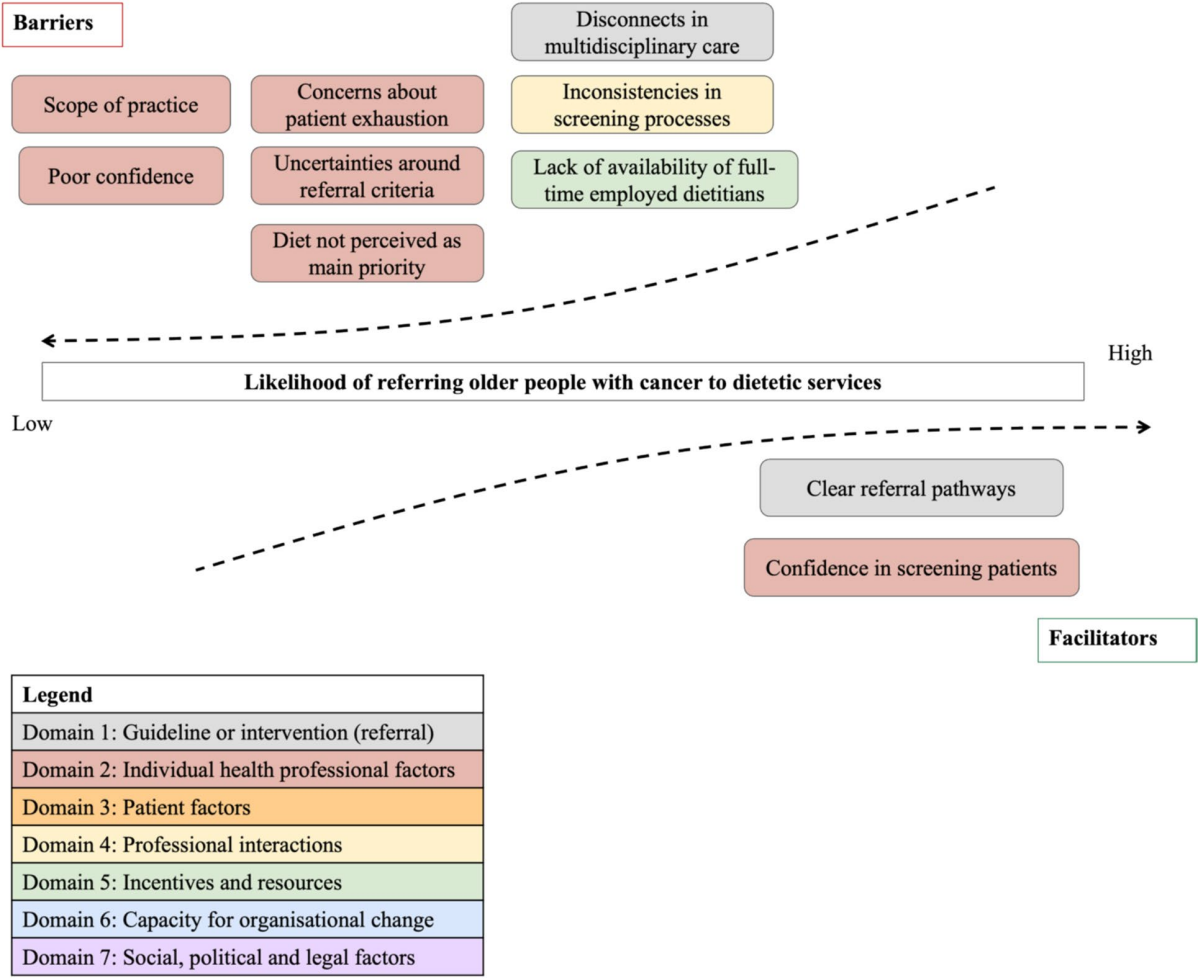
Factors influencing patient referral to dietetic services, categorised into barriers and facilitators and mapped to the relevant TICD domain

Four of the seven TICD domains were identified in relation to factors that influence the referral practices of participants (Fig. 3). The most prominent domain was individual health professional factors (domain 2).

##### Domain 1: Guideline or intervention (referral)

Participants highlighted poor accessibility to dietetic referral pathways, particularly in the outpatient setting, due to the disconnections in multidisciplinary care and a lack of clear and direct referral processes. Conversely, clear referral pathways were described as facilitators.

##### Domain 2: Individual health professional factors

Some participants mentioned concerns and uncertainties related to patients, referral criteria and scope of practice that deterred their referral to dietetics. One participant discussed how his proficiency and confidence in the use of a screening tool facilitated referral.

##### Domain 4: Professional interactions

One participant reported that the inconsistencies in nutritional screening processes within the healthcare system were a major barrier to referral.

##### Domain 5: Incentives and resources

Another participant perceived the poor availability of full-time-employed (FTE) dietitians as a potential barrier to referral.

#### Sub-category 4.2. Barriers and facilitators to the provision of dietary advice

**Fig. 4:**
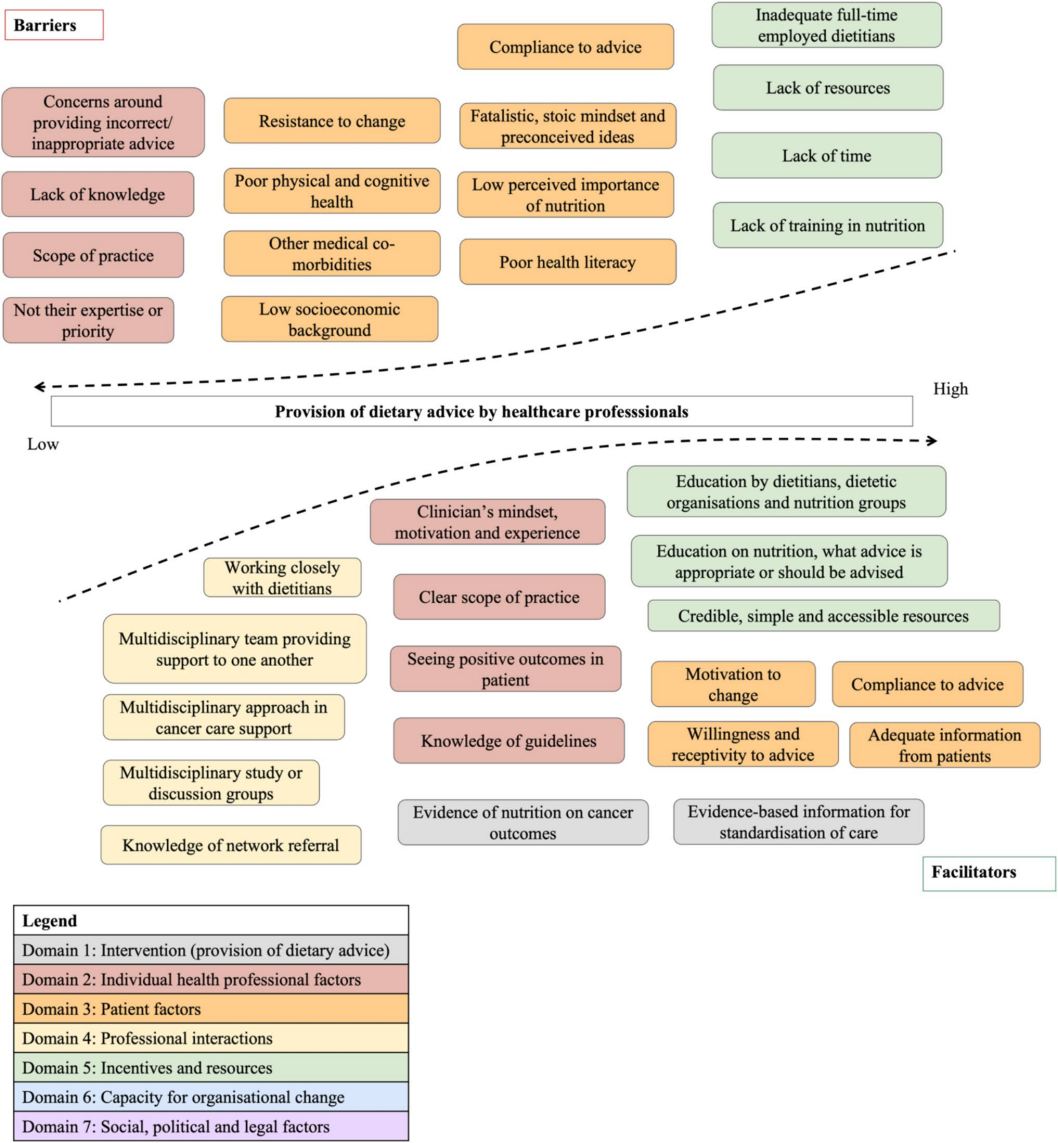
Factors influencing healthcare professionals’ provision of dietary advice to older people with cancer, categorised into barriers and facilitators and mapped to the relevant TICD domains

Five of the seven TICD domains were identified in relation to factors that influence the provision of dietary advice (see Fig. 4). The three prominent domains were individual health professional factors, incentives and resources and patient factors.

##### Domain 1: Intervention

Some participants felt that adequate scientific evidence of nutrition on cancer and health outcomes were important facilitators, and research was perceived as important to standardise the care for older people with cancer.

##### Domain 2: Individual health professional factors

Majority of the participants stressed that nutrition was not their expertise or priority and were often worried and cautious about providing advice outside of their practice scope. Participants desired for better clarity in the roles and responsibilities regarding the provision of dietary advice and the knowledge of dietary guidelines. Many participants highlighted their concerns around providing incorrect or inappropriate advice, and they often attributed these concerns to a perceived lack of nutritional knowledge.

##### Domain 3: Patient factors

The commonly reported patient-related barriers included participants’ perceptions of patients’ preconceived nutritional beliefs and knowledge, resistance to change and low socioeconomic status (Fig. 4). Several participants talked about how a patient’s poor health literacy or low socio-economic status increased the challenge of providing advice. Some participants reported their experiences with older people having a fatalistic mindset about their health. Other participants perceived long-term dietary habits in older adults as difficult to change and felt that patients often did not prioritise nutrition and were more eager to understand their medical treatments. Other perceived barriers closely related to an older person include co-morbidities and poor physical and cognitive health.

##### Domain 4: Professional interactions

Many participants felt that team processes, including a multidisciplinary approach and knowledge of referral networks, would facilitate advice provision as the multidisciplinary team (MDT) can provide support to one another. Some participants discussed how working closely with a dietitian gave them the confidence to provide dietary advice.

##### Domain 5: Incentives and resources

The most reported barrier to the provision of dietary advice across all domains was the lack of time. Most participants mentioned that consultation times were limited and insufficient for them to provide advice, especially individualised advice. One participant mentioned that the lack of FTE dietitians was also a barrier as their role was to reinforce given advice.

Participants also perceived a lack of physical nutrition resources and training. Many participants expressed the need for nutritional education on what advice is appropriate and clarity on when to refer, as well as physical resources. Ideal resources were described as evidence-based and accessible, with simple information catered to an older person. Education by dietetic professionals was also identified as a facilitator.

## Discussion

This study provides comprehensive insights into the perspectives and experiences of non-nutritionally trained HCPs regarding the provision of nutritional care to older people with cancer. To the authors’ knowledge, this is the first study in Australia that examined HCPs’ perceptions regarding their referral practices to dietetics and the provision of dietary advice specifically to older people with cancer. Overall, dietary advice to people with cancer was perceived by participants to be important yet inadequate due to various reasons, including poor access to dietetic services, inconsistencies in referral practices and perceived sub-optimal provision of dietary advice by HCPs other than dietitians. The challenges in providing optimal nutritional care to an older person with cancer are multifaceted.

### Inadequate provision of dietary advice and lack of clarity around perceived role

Most participants perceived that the provision of dietary advice to older people with cancer is inadequate. This study also found that HCPs perceived their own advice to be of sub-optimal quality, and advice provision was not a standard practice. This sentiment is supported by a recent review of patients’ perspectives; cancer survivors in Australia commonly reported limited and ineffective dietary information from HCPs post-cancer treatment [15]. Most participants believed that they had some role to play in the provision of dietary advice; however, there was diversity in responses regarding their specific roles and perceived uncertainty in responsibilities. These findings were mirrored by recent reviews involving HCPs, indicating poor clarification in the scope of practice around the provision of dietary advice [16, 34]. Consequently, patients may not receive adequate nutritional support across their cancer care as the responsibility of providing advice gets shifted from one professional to another [35]. This highlights the importance of clearly defined roles in the provision of dietary advice within a healthcare setting and system. Not all patients have access to a dietitian throughout their cancer care. Hence, it is important to improve the clarity of roles via good communication within each healthcare organisation and to increase HCPs’ awareness of this issue since they are well placed to contribute to general advice provision and reinforcement of the importance of nutrition to older people with cancer.

### Factors influencing the provision of dietary advice by HCPs

Key perceived barriers to providing dietary advice included lack of time, concerns around providing incorrect or inappropriate advice, lack of nutritional knowledge and staying within their scope of practice. Contrastingly, the most represented facilitators were education on nutrition, evidence-based resources and a multidisciplinary approach to cancer care. These factors were similarly reported in reviews looking at HCPs’ perspectives regarding the provision of dietary advice to people with cancer and another expert consensus study that identified essential elements for optimal dietary practices [16, 18, 36].

Interestingly, some barriers in this present study fell under the domain: patient factors, which were not identified in previous reviews [16, 18]. This may mean that there are barriers unique to an older person with cancer, such as the challenges associated with multiple co-morbidities, perceived fatalistic mindset and preconceived ideas around nutrition. This is unsurprising as older people with cancer have distinct nutritional needs as compared to their younger counterparts [37]. Patient-level barriers, including perceived difficulties in changing an older person’s eating habits due to preconceptions around nutrition, were also identified in another study [38].

The diversity of barriers and facilitators mapped to several domains of the TICD checklist suggests that a multi-pronged approach targeting patients, healthcare professionals, professional interactions, incentives and resources is required. Given the specialised needs and potential differences in perspectives of older people, it is worthwhile to examine the views of older people with cancer regarding the receival of dietary advice from HCPs using qualitative methodology. This would allow for a deeper understanding of patient-level factors from the patient’s perspective and the development of appropriate patient-targeted interventions that consider these unique factors. To tackle barriers within the individual health professional domain, there should be a championing of education for HCPs not formally trained in nutrition. Education should focus on what general dietary advice can be provided to older people, guidance on when to provide and when to refer patients to dietitians, as well as dietary guidelines for people with cancer. Nutrition topics should be included within the undergraduate curriculum of non-nutritionally trained HCPs, given the current inadequacy of nutrition education [36]. Other potential educational methods include continuing professional and inter-professional education programs; dietitians and professional dietetic bodies can act as educators for other HCPs. In the context of professional interactions, enhancing collaboration between dietetics and other disciplines would support the delivery of effective dietary advice to people with cancer. Strengthening the multidisciplinary approach in the cancer care pathway is a key facilitator to optimising nutritional care. Lastly, evidence-based resources were highly sought by HCPs; these need to be tailored to an older person’s needs and include advice for where to seek help if they do require more information. The development of such resources and guidelines for HCPs may help them to navigate general advice provision within their limited consultation time.

### Inconsistent referral practices

Although many participants perceived the importance of their role in referring patients to the dietitian, referral was often limited by disconnections in multidisciplinary care and the lack of referral pathways, particularly in the outpatient setting. Disconnections related to poor accessibility of referral pathways and the lack of standardised and structured referral processes were similarly expressed by medical and nursing professionals in a recent Australian review [16]. The implementation of nutrition care pathways, including structured referral systems, has been previously examined in patients with upper gastrointestinal cancer and was associated with improved patient outcomes like lower rates of malnutrition and higher rates of dietetic interventions [40]. The use of evidence-based referral pathways is further supported by Cancer Australia in their ten-year Cancer Plan, which outlined actions to develop an optimal care pathway for older people with cancer, and the recent consensus study that highlighted elements of evidence-based care pathways as essential for optimal referral practices for cancer survivors [36, 41].

Cancer care is conventionally specialist-led, and this traditional care model may contribute to the lack of optimal referral pathways outside of an acute healthcare setting [42]. Given that referral processes would differ across different settings, healthcare transitions need to be improved, and clearer referral pathways may improve access to dietetic services. Referral protocols should include information for HCPs on how, when and where to refer older people with cancer to dietetics. Although the inadequate and delayed referrals of cancer patients have been well-documented, there is limited research on HCPs’ perceived knowledge of and skills in referral and their input on the development of referral pathways for older people with cancer, which should be further explored in future qualitative studies [43]. Intervention trials examining the feasibility and use of referral pathways and protocols may also be warranted.

### Strengths and limitations

One of the strengths of this study was the diversity of the sample, including a range of HCPs, years of work experience and work settings. The use of the TICD checklist strengthened the analysis of barriers and facilitators by organising them into different domains, enhancing the usefulness of findings for other researchers, HCPs and organisations. Although the study included HCPs that were fundamental to cancer care in older adults (e.g. medical oncologist, general practitioner, oncology nurse practitioners, geriatricians, physiotherapist), it did not capture every allied health profession—in particular, point-of-care nurses who seem to be the top referrers to dietitians. Future studies should explore the views of different professions in different countries, as there may be geographical and cultural differences.

## Conclusion

This study adds to the limited literature on HCPs’ perspectives and experiences around referral to dietetics and the provision of dietary advice for older people with cancer, with unique patient factors identified. Future qualitative studies involving older people with cancer are warranted to gain a deeper understanding of patient-level factors influencing the provision of dietary advice by HCPs. Key perceived barriers and facilitators around referral practices and the provision of dietary advice suggest a need for a multi-pronged approach to improve the nutritional care of older adults with cancer. Simple and accessible nutrition resources, advocacy for nutrition education of HCPs and improved dietetic referral pathways are required to better support a multi-disciplinary approach in nutritional care.

## Supplementary Information

Below is the link to the electronic supplementary material.

Supplementary file1 (DOCX 1593 KB)

## Data Availability

Data is provided within the manuscript or supplementary information files.
