# Supplementary material for: A qualitative descriptive study on the perspectives and experiences of multidisciplinary HCPs in providing nutritional care to older adults with cancer
Source: Support Care Cancer. 2025 Feb 25;33(3):221. doi: 10.1007/s00520-025-09254-7 (PMC11861013; doi:10.1007/s00520-025-09254-7)
Supplement: Supplementary file 1 — Supplementary file1 (DOCX 1593 KB) [file 520_2025_9254_MOESM1_ESM.docx]

Supplementary Material 1: Philosophical and theoretical approaches of this study

Supplementary Material 2: TICD checklist


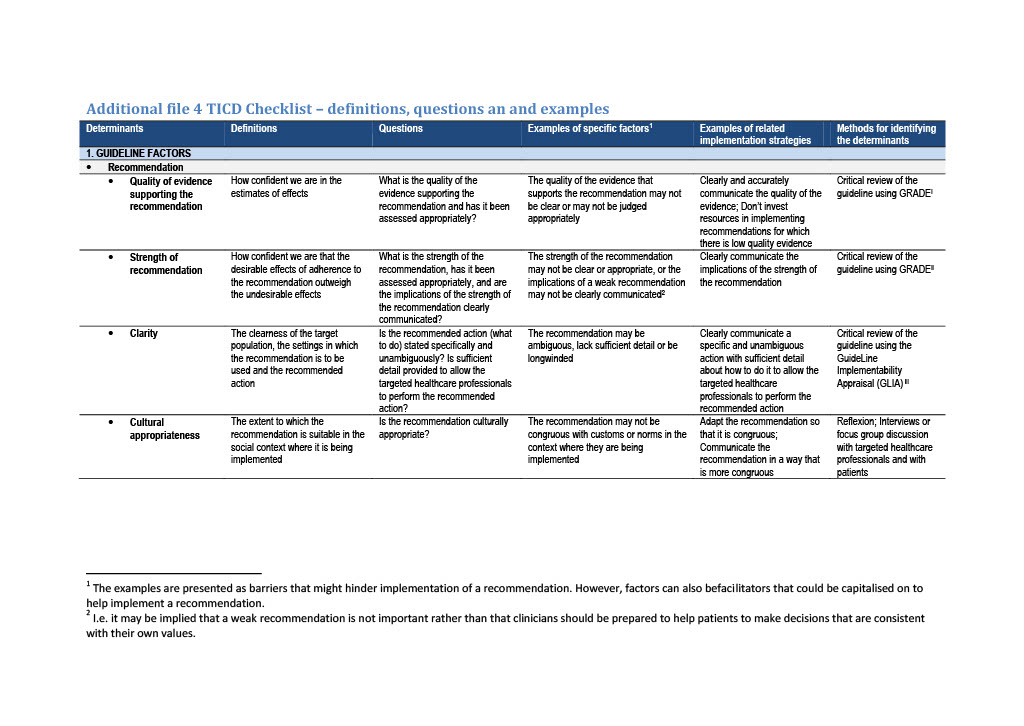

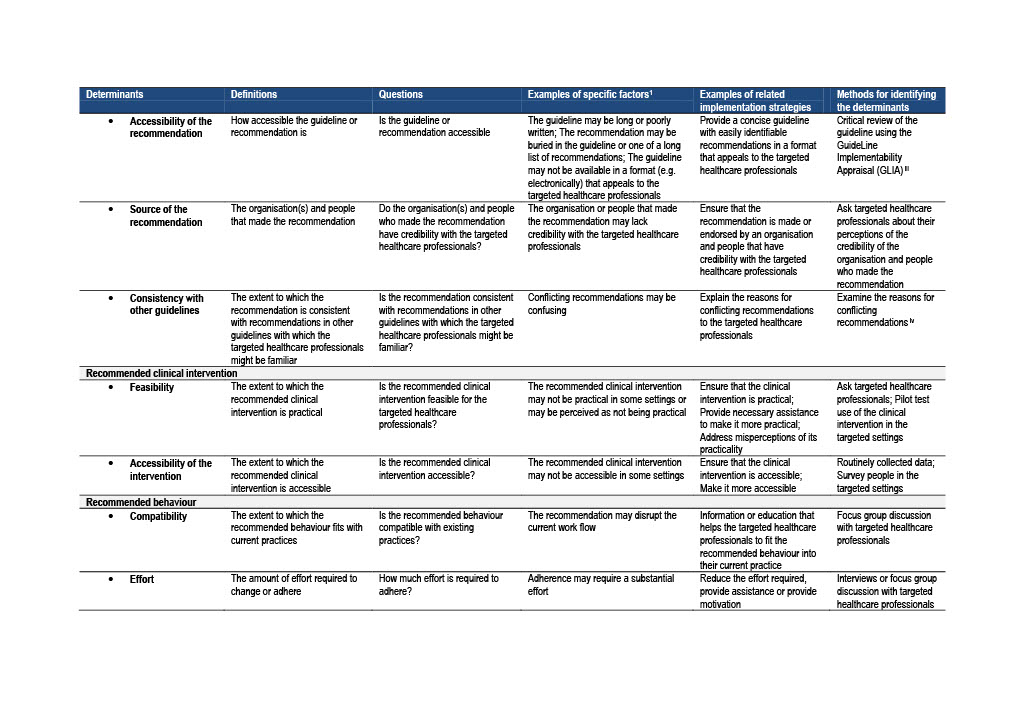

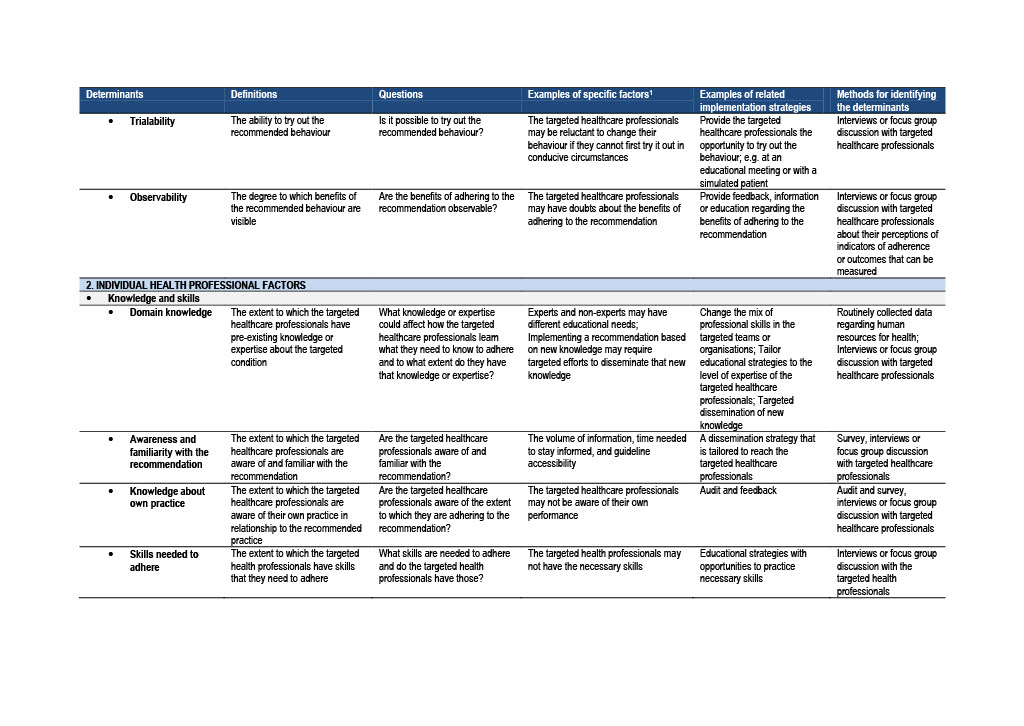

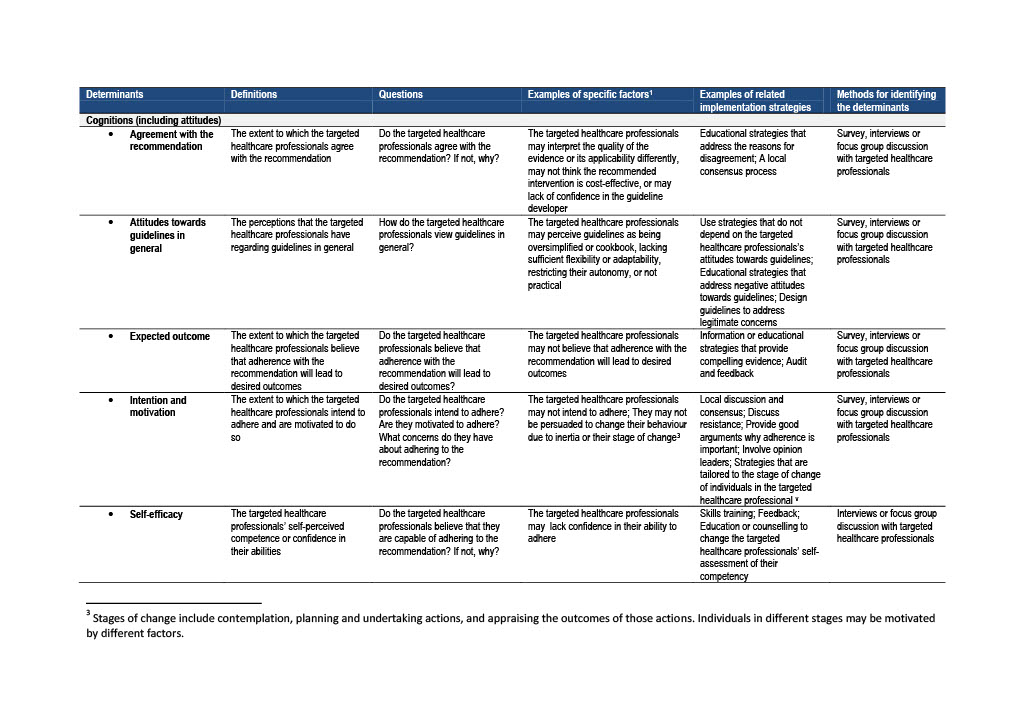

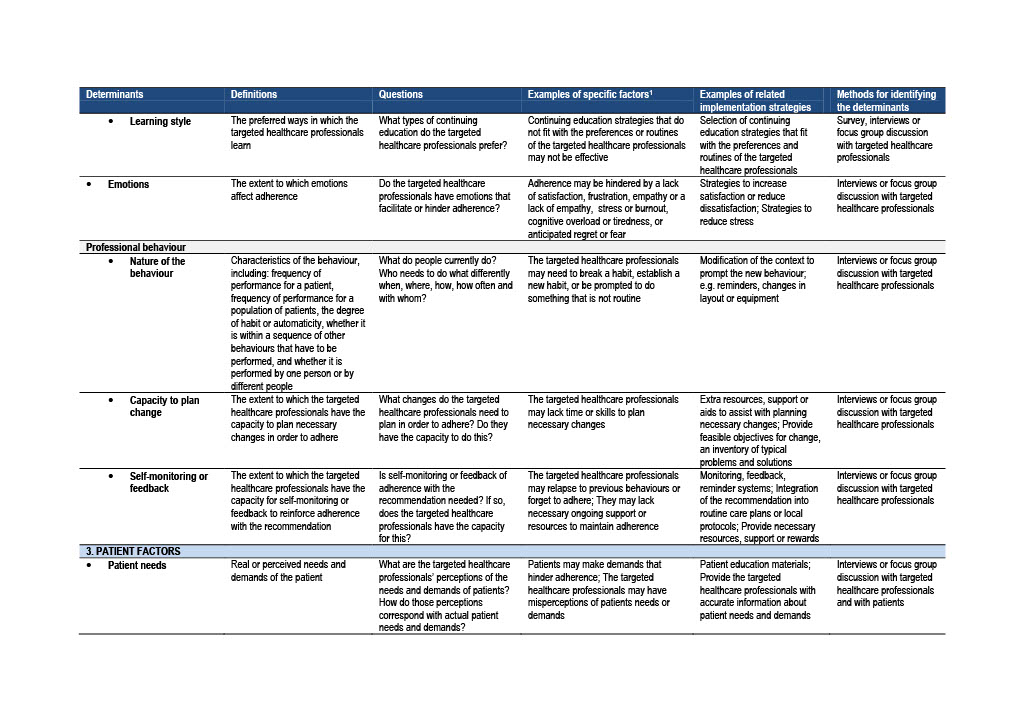

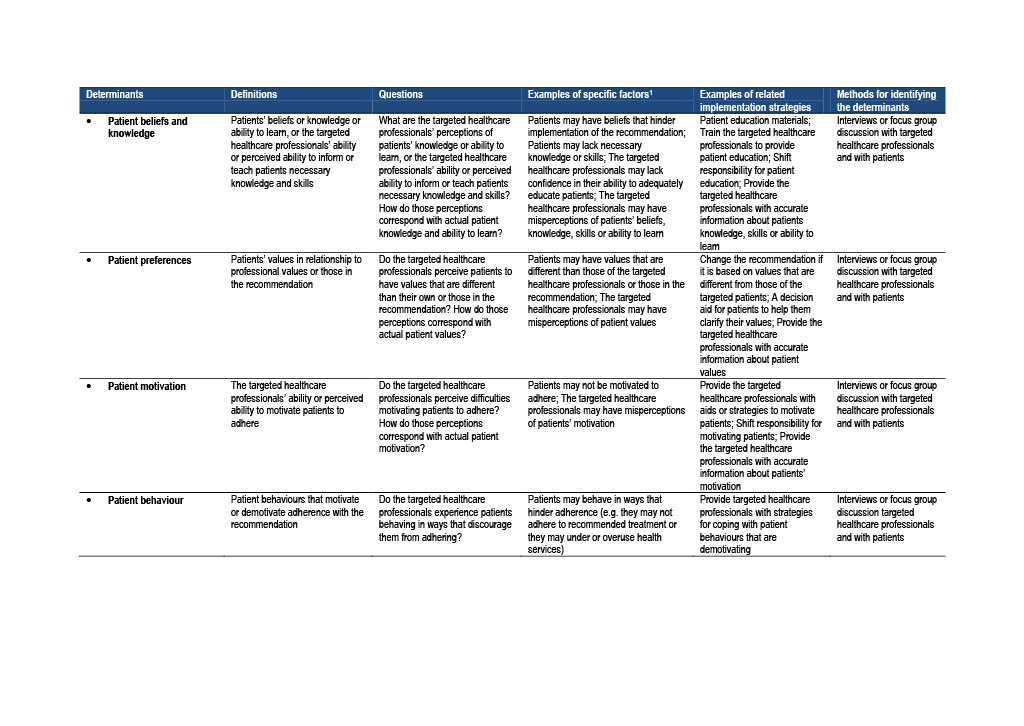

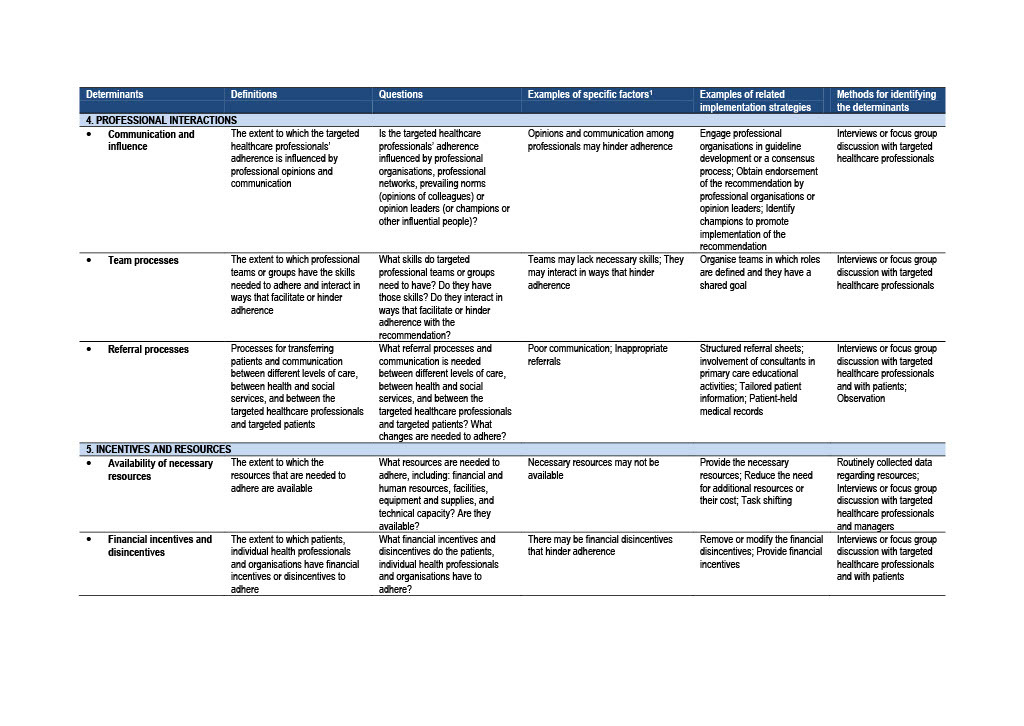

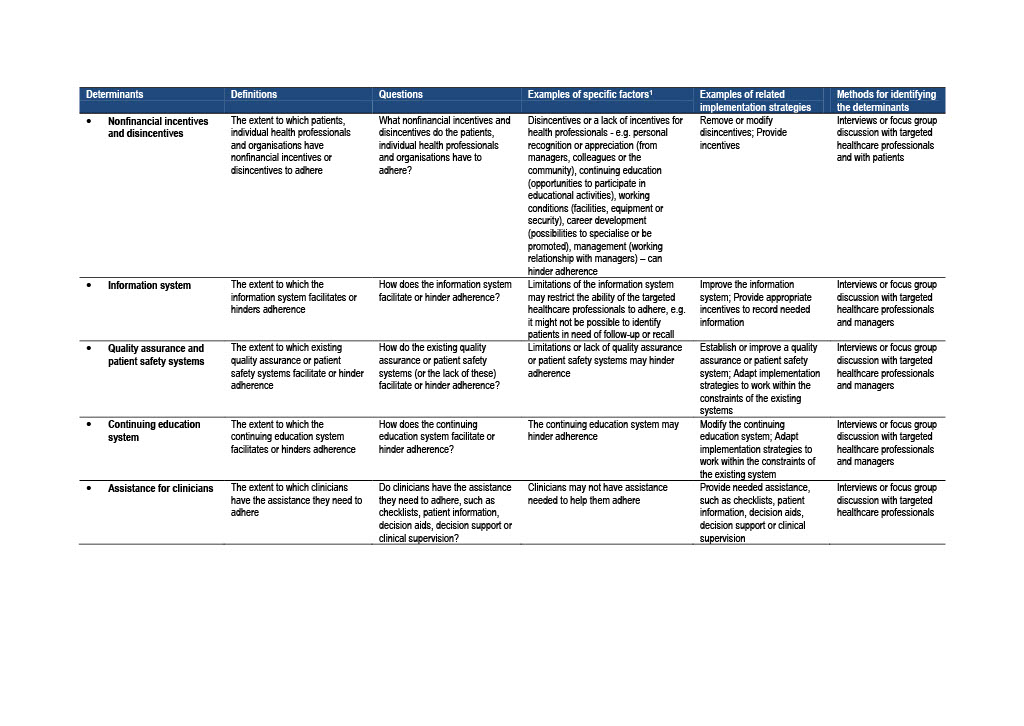

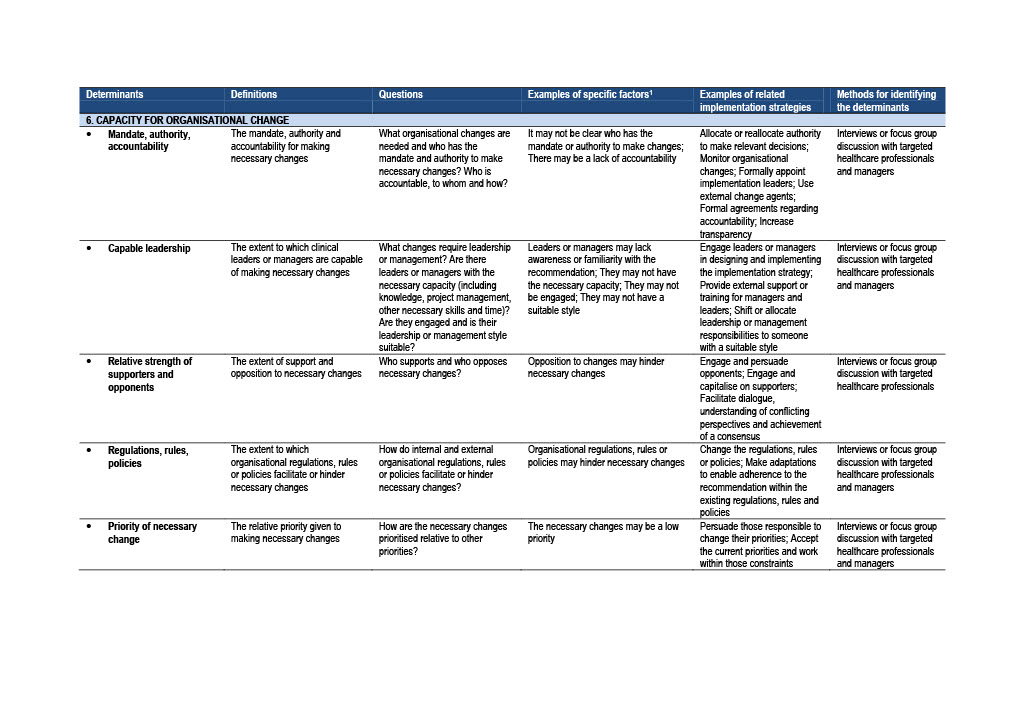

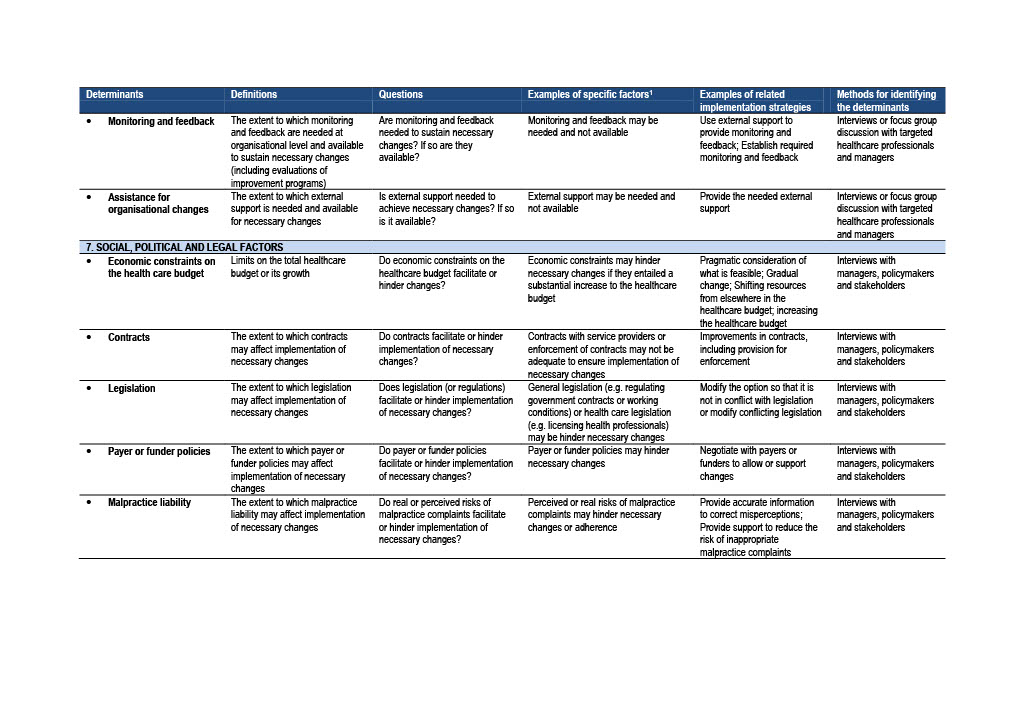

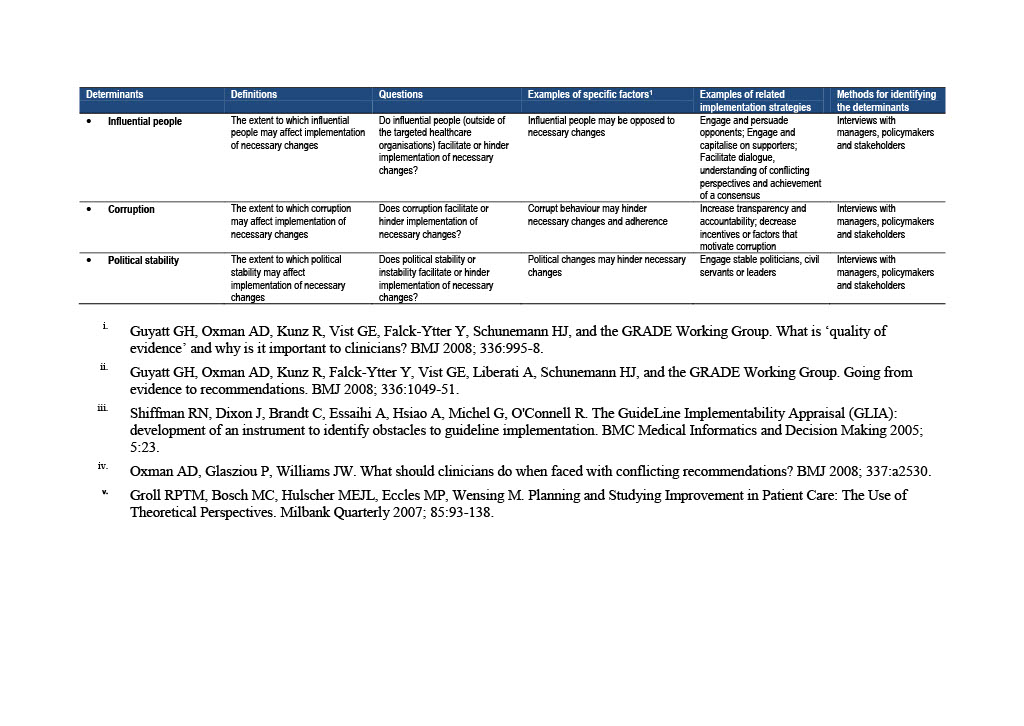


Reference:

Flottorp SA, Oxman AD, Krause J, et al. A checklist for identifying determinants of practice: A systematic review and synthesis of frameworks and taxonomies of factors that prevent or enable improvements in healthcare professional practice. *Implementation Science.* 2013;8(1):35. <https://doi.org/10.1186/1748-5908-8-35>

Supplementary Material 3**:** Semi-structured interview guide

**Section 1: Participant information/ building rapport**

- What is your current profession/role? E.g., nurse, medical oncologist etc.
- How long have you been providing care to people with cancer?
- What setting do you work in? e.g., community/hospital etc.
- What proportion of your work time is spent working with people with cancer?
- What is your current role/position and how regularly do you consult with older people with cancer?

**Section 2: Their understanding around the role of dietary advice**

1. What is your understanding of the relationship between nutrition and diet and health-related and cancer outcomes in older people with cancer, including the prevalence and importance of (mal)nutrition in older people with cancer on e.g. quality of life, mortality etc.
2. Do you think it is important for older people with cancer to be provided with adequate nutritional advice/education/care? Why/why not?

**Section 3: Asking specific questions on their perspectives of providing dietary advice to older people with cancer**

1. What do you think is your role in the provision of nutritional advice/education/care? Explain.
2. Do you provide older patients with nutritional advice/education/care? What type? How often?
3. Do you refer your patients to dietetics for nutritional advice/education/care? When? Why?
4. Are you aware of nutritional/dietary guidelines for people with cancer? Do you follow them?

**Section 4: Any experiences they would like to share.**

1. Have you experience any barriers to providing nutritional advice/education/care?
2. What do you think has helped you in providing nutrition advice/ education?
3. Are you aware of perceptions regarding provision of nutritional advice/education/care experienced by other health care providers involved in cancer care?
4. Do you think that nutritional advice/ education is adequate for older people with cancer? Why/why not?

**Wrapping up:**

1. Do you have any other comments? Or is there anything else that we should have talked about but did not touch on?

Thank you very much for your time.

Supplementary Material 4**:** Coding framework

| **Category 1:** HCPs’ perceived importance of nutrition in cancer care | |
| --- | --- |
| Sub-category | Codes |
| 1.1 Awareness and perceptions of the role of nutrition | Huge role in health and cancer-related outcomes |
|  | Awareness of high risk of malnutrition in patients with cancer, especially for older people |
|  | Relationship between nutrition and overall health and cancer outcomes (good nutrition) |
|  | Relationship of nutrition and cancer is overrated |
| 1.2 Perceived importance of adequate dietary advice | It is important for older people to be provided with adequate dietary advice |
|  | It is important as part of comprehensive supportive care |
|  | The importance of adequate dietary advice depends on the goal of patients |

| **Category 2:** HCP's perceived role in nutritional care | | |
| --- | --- | --- |
| Sub-category | Condensed codes | Codes |
| 2.1  Perceived role in the provision of dietary advice | Diversity on professional role in providing dietary advice: It is everyone's role to a certain extent | Their role is to provide general basic advice within their scope of practice and directing patients to relevant information |
|  |  | Their role is to provide advice specific to their specialty |
|  |  | There is opportunity for blended roles and it should be part of their responsibility |
|  |  | Their role is to support the dietitian or the work of the dietitian |
|  | The dietitian is the most qualified person to provide dietary advice | Dietitian is the expert |
| 2.2  Perceived role in referring patients to the dietitian |  | Steering patients to the right person or linking them to the dietitian |
| 2.3  Perceived role in screening patients who require dietetic services |  | Identifying problems early |
|  |  | Picking up red flags |
|  |  | Supporting the dietitian's role by screening |

| **Category 3:** Current provision of nutritional care to older people with cancer | | |  |
| --- | --- | --- | --- |
| Sub-category | Condensed codes | Codes |  |
| 3.1  Referral practices for older people with cancer to dietetic services | Reasons or criteria for referral to dietetics | Concerns about weight and malnutrition |  |
|  |  | Outside of scope of practice (provision of specific advice) |  |
|  | Referral processes in different settings | Informal methods |  |
|  |  | Formal referral systems |  |
|  |  | Team effort and responsibility |  |
| 3.2  Perceived inadequacy of current provision of dietary advice | Current dietary advice provided is inadequate due to various reasons | Missed referrals or screening |  |
|  |  | Diet is not the priority |  |
|  |  | Poor accessibility |  |
|  |  | Lack of trained professions |  |
|  |  | Multi-morbidities in older people |  |
|  |  | Patients not on treatment |  |
|  | Dietary advice is only adequate when patients see a dietitian | Advice is adequate if there is dietetics intervention |  |
| 3.3  Perspectives on their own provision of dietary advice | Quality of advice | Not the best |  |
|  |  | Advice provided is general and basic and informal |  |
|  | Different sources of inform their knowledge | Education |  |
|  |  | Drug companies |  |
|  |  | Past work experiences |  |
|  | Frequency of dietary advice provision | Frequency of advice provision varied amongst HCP; nurses and oncologists may provide more advice |  |
|  |  | Nutrition generally not part of their consultations |  |
| 3.4  Current awareness knowledge, and use of evidence-based sources | Use of evidence-based resources when providing advice | A number of participants used resources that are evidence-based |  |
|  | Use of dietary guidelines | Poor knowledge, awareness and use of dietary guidelines for people with cancer |  |

| **Category 4:** Factors influencing the provision of optimal nutritional care  *Sub-category 4.1. Barriers and facilitators to patient referral to dietetic services* | | | |
| --- | --- | --- | --- |
| Condensed codes | Codes | TICD domain | TICD determinant |
| Barriers and challenges associated with referral of patient to dietetic services | No confidence in referring patients | Individual health professional factors | Self-efficacy |
|  | Diet was not perceived as the main priority for patients | Individual health professional factors | Intention and motivation |
|  | Scope of practice perceived to be not related to nutrition | Individual health professional factors | Nature of behaviour (not area of expertise) |
|  | Uncertainties around referral criteria | Individual health professional factors | Knowledge and skills around referring |
|  | Concerns about patient exhaustion with multiple appointments | Individual health professional factors | Emotions related to referring patients Expected outcome |
|  | Disconnects in multidisciplinary care due to lack of direct referral pathways | Guideline/ intervention factors | Accessibility of referral pathways |
|  | Inconsistencies in screening processes | Professional interactions | Referral processes Team processes |
|  | Lack of availability of FTE dietitians | Incentives and resources | Availability of necessary resources |
| Facilitators to patient referral to dietetic services | Clear referral pathways | Guideline/ intervention factors | Accessibility Effort |
|  | Confidence in screening patients | Individual health professional factors | Self-efficacy |

| **Category 4:** Factors influencing the provision of optimal nutritional care  *Sub-category 4.2.* *Barriers and facilitators to provision of dietary advice* | | | |
| --- | --- | --- | --- |
| Condensed codes | Codes | TICD domain | TICD determinant |
| Barriers to provision of dietary advice | Concerns around providing advice outside of their scope | Individual health professional factors | Nature of the behaviour (scope of practice) |
|  | Concerns around providing incorrect or inappropriate advice | Individual health professional factors | Domain knowledge |
|  | Lack of knowledge | Individual health professional factors | Domain knowledge |
|  | Not their expertise or priority | Individual health professional factors | Knowledge and skills |
|  | Compliance | Patient factors | Compliance |
|  | Patient's resistance to change due to various reasons | Patient factors | Patient motivation |
|  | Patient's capability and cognition | Patient factors | NA |
|  | Knowledge or perceived importance of nutrition | Patient factors | Patient beliefs and knowledge |
|  | Patient's mindset or preconceived ideas | Patient factors | Patient beliefs and knowledge |
|  | Poor health literacy | Patient factors | Patient beliefs and knowledge |
|  | Socio-economic factors | Patient factors | NA |
|  | Complications in patient's needs | Patient factors | Patient needs |
|  | Inadequate FTE dietitians | Incentives and resources | Availability of necessary resources |
|  | Lack of resources | Incentives and resources | Availability of necessary resources |
|  | Lack of time | Incentives and resources | Availability of necessary resources |
|  | Lack of training in nutrition | Incentives and resources | Continuing education system |
| Facilitators to the provision of dietary advice | Importance of a multidisciplinary approach in supporting patients with cancer | Professional interactions | Team processes |
|  | Multidisciplinary study or discussion groups | Professional interactions | Team processes |
|  | Knowledge of referral networks gives confidence in provision of advice | Professional interactions | Team processes |
|  | Working closely with dietitians gives confidence to provide advice | Professional interactions | Communication and influence |
|  | A multidisciplinary team provides support to one another | Professional interactions | Communication and influence |
|  | Patient compliance to advice | Patient factors | Compliance |
|  | Having adequate information from patients | Patient factors | Patient behaviour |
|  | Patient's motivation to change | Patient factors | Patient motivation |
|  | Patient's willingness and receptivity to dietary advice | Patient factors | Patient behaviour |
|  | Credible resources (evidence-based) | Incentives and resources | Availability of necessary resources |
|  | Simple resources | Incentives and resources | Availability of necessary resources |
|  | Accessible resources | Incentives and resources | Availability of necessary resources |
|  | Evidence of nutrition on cancer outcomes promotes involvement in dietary advice and care | Guideline/ intervention factors | Quality of evidence supporting recommendation |
|  | Evidence-based information for standardisation of nutritional care | Guideline/ intervention factors | Quality of evidence supporting recommendation |
|  | Clear scope of practice (knowing their role) | Individual health professional factors | Scope of practice |
|  | Clinician's mindset, motivation, and experience | Individual health professional factors | Intention and motivation |
|  | Seeing positive outcomes | Individual health professional factors | Expected outcome |
|  | Knowledge of guidelines | Individual health professional factors | Knowledge Awareness and familiarity with recommendations |
|  | Education by dietitians, dietetic organisations, or nutrition groups | Incentives and resources | Continuing education system |
|  | Education on nutrition, what advice is appropriate or should be advised | Incentives and resources | Continuing education system |

Supplementary Material 5: Standard for Reporting Qualitative Research (SQRQ) Checklist

| **Title and abstract** | | **Page/line no(s).** |
| --- | --- | --- |
|  | **Title** - Concise description of the nature and topic of the study Identifying the study as qualitative or indicating the approach (e.g., ethnography, grounded theory) or data collection methods (e.g., interview, focus group) is recommended | Page 1, Line 3-5 |
|  | **Abstract** - Summary of key elements of the study using the abstract format of the intended publication; typically includes background, purpose, methods, results, and conclusions | Page 2, Line 19-46 |
|  |  |  |
| **Introduction** | |  |
|  | **Problem formulation** - Description and significance of the problem/phenomenon studied; review of relevant theory and empirical work; problem statement | Page 3-4 Line 47-86 |
|  | **Purpose or research questio**n - Purpose of the study and specific objectives or questions | Page 4, 79-86 |
|  |  |  |
| **Methods** | |  |
|  | **Qualitative approach and research paradigm** - Qualitative approach (e.g., ethnography, grounded theory, case study, phenomenology, narrative research) and guiding theory if appropriate; identifying the research paradigm (e.g., postpositivist, constructivist/ interpretivist) is also recommended; rationale** | Page 5, Line 88-102  + Supplementary material 1 and 2 |
|  | **Researcher characteristics and reflexivity** - Researchers’ characteristics that may influence the research, including personal attributes, qualifications/experience, relationship with participants, assumptions, and/or presuppositions; potential or actual interaction between researchers’ characteristics and the research questions, approach, methods, results, and/or transferability | Page 9, Line 153-163 |
|  | **Context** - Setting/site and salient contextual factors; rationale** | Page 6, Line 106-118 |
|  | **Sampling strategy** - How and why research participants, documents, or events were selected; criteria for deciding when no further sampling was necessary (e.g., sampling saturation); rationale** | Page 6, Line 103-115 |
|  | **Ethical issues pertaining to human subjects** - Documentation of approval by an appropriate ethics review board and participant consent, or explanation for lack thereof; other confidentiality and data security issues | Page 6, Line 117-124 |
|  | **Data collection methods** - Types of data collected; details of data collection procedures including (as appropriate) start and stop dates of data collection and analysis, iterative process, triangulation of sources/methods, and modification of procedures in response to evolving study findings; rationale** | Page 6-7, Line 126-148 |
|  | **Data collection instruments and technologies** - Description of instruments (e.g., interview guides, questionnaires) and devices (e.g., audio recorders) used for data collection; if/how the instrument(s) changed over the course of the study | Page 6-7, Line 126-134 |
|  | **Units of study** - Number and relevant characteristics of participants, documents, or events included in the study; level of participation (could be reported in results) | Table 1 |
|  | **Data processing** - Methods for processing data prior to and during analysis, including transcription, data entry, data management and security, verification of data integrity, data coding, and anonymization/de-identification of excerpts | Page 7, Line 136-148 |
|  | **Data analysis** - Process by which inferences, themes, etc., were identified and developed, including the researchers involved in data analysis; usually references a specific paradigm or approach; rationale** | Page 7, Line 136-148 |
|  | **Techniques to enhance trustworthiness** - Techniques to enhance trustworthiness and credibility of data analysis (e.g., member checking, audit trail, triangulation); rationale** | Page 9, Line 157-164 |
|  |  |  |
| **Results/findings** | |  |
|  | **Synthesis and interpretation** - Main findings (e.g., interpretations, inferences, and themes); might include development of a theory or model, or integration with prior research or theory | Page 10-19, Line 164-318  Table 1, Figure 2, Supplementary Material 6 |
|  | **Links to empirical data** - Evidence (e.g., quotes, field notes, text excerpts, photographs) to substantiate analytic findings | Supplementary Material 6 (quotes)  Supplementary Material 4 (coding framework) |
|  |  |  |
| **Discussion** | |  |
|  | **Integration with prior work, implications, transferability, and contribution(s) to the field -** Short summary of main findings; explanation of how findings and conclusions connect to, support, elaborate on, or challenge conclusions of earlier scholarship; discussion of scope of application/generalizability; identification of unique contribution(s) to scholarship in a discipline or field | Page 20-23, Line 319-414 |
|  | **Limitations** - Trustworthiness and limitations of findings | Page 22-23, Line 399-406 |
|  |  |  |
| **Other** | |  |
|  | **Conflicts of interest** - Potential sources of influence or perceived influence on study conduct and conclusions; how these were managed | Page 24, Line 435-6 |
|  | **Funding** - Sources of funding and other support; role of funders in data collection, interpretation, and reporting | Page 24, Line 426-7 |

Reference:

O'Brien BC, Harris IB, Beckman TJ, Reed DA, Cook DA. Standards for reporting qualitative research: a synthesis of recommendations. *Acad Med*. 2014;89(9):1245-1251. doi: [10.1097/ACM.0000000000000388](https://doi.org/10.1097/acm.0000000000000388)

Supplementary Material 6: List of representative participant quotes relevant to each sub-category

| **Sub-category 1.1.** Awareness and perceptions of the role of nutrition | “*…talking to patients about the importance of nutrition when they're having cancer treatment… they have higher nutritional needs while they're going through cancer therapy…”* (P3, Nurse practitioner)  *“Do I think that patients cancer outcomes depend on their diet? Well, I haven't yet to see any evidence to say that, but there's possibly a more nuanced approach. Because, you know is diet important? Yes, that's what we eat. Yes, but doesn't affect the outcomes from their cancers*… *and it (nutrition) forms part of comprehensive supportive care process*” (P18, Oncologist).  “*I think if you're not well fuelled… it just makes sense that their outcomes can be poor in terms of how they tolerate treatment…*” (P8, Physiotherapist) |
| --- | --- |
| **Sub-category 1.2.** Perceived importance of adequate dietary advice | “*…I think it’s part of the multidisciplinary support… and if they want to control through their diet, that's a positive thing*” (P18, Oncologist)  “*depends on what the goal of the nutritional advice is. So, if we are trying to cure them, then yes…*” (P4, Oncologist) |
| **Sub-category 2.1.** Perceived role in the provision of dietary advice | “*everyone’s role to a certain extent*” (P16, oncologist).  “*…provide advice in line with guideline-based recommendations…*” (P11, Exercise physiologist)  *“…we’ll provide patients with information about MCT diets for instance…”* (P5, Lymphedema specialist)  “*But there is opportunities for blended roles. Rather, you know that if an OT is going out and doing a home assessment around, I don't know their safety in the bathroom or their ability to cook a meal, then then it's a prime opportunity to ask questions related to their diet, their nutrition and their concerns they have to then flag, you know, to provide advice in the context of doing something like that.” (*P6, Occupational therapist). |
| **Sub-category 2.2.** Perceived role in referring patients to dietetic services | “*So I try to just give the basics that I know… it's not my area of expertise… it is also our role to refer to the to the experts, which is the dietitian*.” (P17, Geriatric Oncologist) |
| **Sub-category 2.3.** Perceived role in screening patients who require dietetic services | “*…our roles are really to be aware of how important nutrition and advice plays, but then also pick up any red flags*.” (P6, Occupational therapist) |
| **Sub-category 3.1.** Current referral practices to dietetic services | “*…if I detect loss of weight, poor nutrition, sarcopenia, elements of malnutrition, I will 100% refer to a dietician… It's beyond my scope sometimes… therefore, I outsource and get the specialist (dietitian) involved…*” (P17, Geriatric oncologist)  *“But we're very good in our hospital because the nurses detect it too because they did they they weigh patients and things like this. So if the support care nurses or any of the nurses detect that they need a dietician, they can also refer so we can refer not just by doctors, we can refer by other clinicians.”* (P18, Oncologist)  “*The nurses do the referrals*” (P2, Oncologist). |
| **Sub-category 3.2.** Perceived inadequacy of the current provision of dietary advice | “*…because older people have so much complexity with multi conditions*” (P6, Occupational therapist)  “*…if they're not on any treatment, the advice is probably close to nothing unless the patient has requested to see a dietitian…*” (P4, Oncologist)  “*I think if they're seeing a dietitian, it's really, really good…*” (P8, Physiotherapist). |
| **Sub-category 3.3.** Perspectives on their own provision of dietary advice | “…*certainly our nurses in our chemo suite would be talking to patients quite frequently about… general advice around nutrition*” (P3, Nurse practitioner)  “*Simple is good… I think I don’t specify anything more so than that.*” (P15, Audiologist)  “*I would not say that it's optimal or the best evidence-based by any stretch*” (P16, Oncologist)  “*It could be from past experience… sometimes it’s a group course for education…*” (P13, Pharmacist) |
| **Sub-category 3.4.** Current awareness, knowledge and use of evidence-based sources | “*…as far as I’m aware, there’s no specific guidelines for patients*” (P14, Oncologist) “*And a lot of that information comes from outside sources, such as the Cancer Council*.” (P3, Nurse practitioner) |
| **Sub-category 4.1.** Barriers and facilitators to patient referral to dietetic services by HCPs | Domain 1: Guideline or intervention (referral)  “*…it's just hard to facilitate that (multidisciplinary care) in a really busy public hospital*” (P3, Nurse practitioner).  “*a common place to find sites where we can refer patients to that would make life a lot easier*” (P11, Exercise physiologist)**.**  Domain 2: Individual health professional factors  “*…is something to be alarming to trigger our referral or if it's going to be burdensome for their caseload that day?... I wouldn't be as confident with that referral*.” (P7, Speech pathologist)  “*…I think I'm quite confident in doing that (screening)… we do have a screening tool that we can use called MUST which the dietitians would like us to use as part of the referral…*” (P3, Nurse practitioner)  Domain 4: Professional interactions  “*Even if they are malnourished or not, we don't know, and they will be simply missed… because we are not screening them…*” (P19, General medicine physician).  Domain 5: Incentives and resources  “*maybe like oncology as a team like Allied Health is better at cross-referring than say if you had an older person with cancer that came in under a Gen Med bed… because there's less FTE like patients wouldn't get seen*.” (P9, Physiotherapist). |
| **Sub-category 4.2.** Barriers and facilitators to provision of dietary advice | Domain 1: Intervention  “*… we're really trying to maximise their nutrition outcomes because we have really good evidence in terms of them*” (P3, Nurse practitioner).  Domain 2: Individual health professional factors  “*…inappropriate for us to provide any dietary advice, especially if it's outside of our scope*” (P2, Palliative care specialist)  “*…working through with different professions, what's within their scope of practice, what can any profession do and when is that line when more specialist inputs required*” (P6, Occupational therapist)  Domain 3: Patient factors  “*…the health literacy and the buy in to how important it is and can be tricky*” (P10, Speech pathologist)  “*The socioeconomic background here, I would say medium to low… Can you add this, add that to your diet? To us, it's just saying it, to them, it's probably they need to buy more food. So money can be an issue*” (P12, Nurse practitioner)  “*Or just their age playing into it too, like ‘ohh, that's what happens when you get older’*” (P7, Speech pathologist)  “*… ‘Talk to me about the medicine options’ … the importance of diet and exercise seems less important to them at that moment…*” (P16, Oncologist)  “*… older people… more likely to have other medical comorbidities… try to assess one thing in an older person is a bit more challenging and nutrition is a small part of a big picture*” (P3, Nurse practitioner)  Domain 4: Professional interactions  “*It probably goes back to my relationship with our dietetics team for those high-risk patients… we've established a really good sort of collaborative relationship*” (P3, Nurse practitioner)  Domain 5: Incentives and resources  “*…I'm not gonna have the time to spend an hour with the patient to go through a full diet history*” (P20, General practitioner)  “*I probably found initially like where to find good reliable information that I can provide my clients with was challenging*” (P11, Exercise physiologist)  “*If I was to have more education or, like, knew a little bit more about what we could say…*” (P9, Physiotherapist)  “*I would love an information sheet that's in simple maybe pictures because you know that generation. They don't want to see that many writings…*” (P12, Nurse practitioner)  *“…because we can't reinforce what's not introduced*” (P8, Physiotherapist). |
